# An Analysis of Physiology; Being a Condensed View of the Most Important Facts and Doctrines

**Published:** 1852-08

**Authors:** 


*An analysis of Physiology; being a condensed view of the most important facts and doctrines. Designed especially for the use of students.* By JOHN J. REESE, M. D., Lecturer on Materia Medica and Therapeutics, in the Medical Institue of Philadelphia, &c. &c. Second Edition, revised and enlarged. Philadelphia, Lindsay & Blakiston.

The present edition of Dr. Reese's convenient analysis, has been remodelled by the author, and much of it rewritten; and all that has transpired worthy of note, since the previous issue, has been introduced. As a faithful digest, therefore, it cannot fail to be extremely useful to the student, to whom we cordially recommend it.

